# Superoxide Dismutase 1 and tgSOD1^G93A^ Mouse Spinal Cord Seed Fibrils, Suggesting a Propagative Cell Death Mechanism in Amyotrophic Lateral Sclerosis

**DOI:** 10.1371/journal.pone.0010627

**Published:** 2010-05-13

**Authors:** Ruth Chia, M. Howard Tattum, Samantha Jones, John Collinge, Elizabeth M. C. Fisher, Graham S. Jackson

**Affiliations:** 1 Department of Neurodegenerative Disease, University College London Institute of Neurology, London, United Kingdom; 2 MRC Prion Unit, University College London Institute of Neurology, London, United Kingdom; Brigham and Women's Hospital, Harvard Medical School, United States of America

## Abstract

**Background:**

Amyotrophic lateral sclerosis (ALS) is a neurodegenerative disease that specifically affects motor neurons and leads to a progressive and ultimately fatal loss of function, resulting in death typically within 3 to 5 years of diagnosis. The disease starts with a focal centre of weakness, such as one limb, and appears to spread to other parts of the body. Mutations in superoxide dismutase 1 (SOD1) are known to cause disease and it is generally accepted they lead to pathology not by loss of enzymatic activity but by gain of some unknown toxic function(s). Although different mutations lead to varying tendencies of SOD1 to aggregate, we suggest abnormal proteins share a common misfolding pathway that leads to the formation of amyloid fibrils.

**Methodology/Principal Findings:**

Here we demonstrate that misfolding of superoxide dismutase 1 leads to the formation of amyloid fibrils associated with seeding activity, which can accelerate the formation of new fibrils in an autocatalytic cascade. The time limiting event is nucleation to form a stable protein “seed” before a rapid linear polymerisation results in amyloid fibrils analogous to other protein misfolding disorders. This phenomenon was not confined to fibrils of recombinant protein as here we show, for the first time, that spinal cord homogenates obtained from a transgenic mouse model that overexpresses mutant human superoxide dismutase 1 (the TgSOD1^G93A^ mouse) also contain amyloid seeds that accelerate the formation of new fibrils in both wildtype and mutant SOD1 protein in vitro.

**Conclusions/Significance:**

These findings provide new insights into ALS disease mechanism and in particular a mechanism that could account for the spread of pathology throughout the nervous system. This model of disease spread, which has analogies to other protein misfolding disorders such as prion disease, also suggests it may be possible to design assays for therapeutics that can inhibit fibril propagation and hence, possibly, disease progression.

## Introduction

Amyotrophic lateral sclerosis (ALS) is a neurodegenerative disease defined by the selective death of upper and lower motor neurons [1], [2]. Approximately 10–20% of all ALS cases are familial (fALS), with the remainder idiopathic in origin and defined as sporadic (sALS). Superoxide dismutase 1 (SOD1) is an antioxidant protein expressed abundantly and ubiquitously [3] and since the discovery in 1993 that mutations in SOD1 cause ALS [4], [5] a further 140 mutations have since been found scattered throughout the 153 amino acid Cu/Zn-homodimer. Mutations are mostly substitutions of single amino acid residues although some deletions, insertions and C-terminal truncations have been identified. SOD1 mutations are known to be causative in 10–20% fALS cases, and have been observed in 2% sporadic cases [1].

The presence of inclusion bodies or aggresomes is one of the neuropathological hallmarks of ALS. Spinal cord sections from SOD1-fALS patients have been examined by immunohistochemistry and found to contain aggregates that are reactive to anti-SOD1 antibodies [6]. More recent evidence has shown that in non-SOD1 ALS (fALS and sALS), aggregates also contain SOD1 [7], [8], [9] and there is no marked difference in disease presentation and progression, either clinically or neuropathologically, between fALS and sALS [10].

ALS presents as a neurodegenerative disease beginning as a focal weakness and atrophy of proximal limbs or body region and progressively spreads to distal muscle groups over time [11]. Loss of motor function appears to affect proximal motor units closest to onset site, followed by more distal motor units and this correlates well with the observed gradient loss of motor neurons from the onset site [12]. Clinical disease usually starts abruptly, affecting mostly patients in their mid-life, and has a rapid disease progression (3–5 years) ultimately leading to death, usually due to respiratory failure. This pattern of disease progression suggests some form of cell-to-cell transmission of a toxic ‘factor’, where morbid or dying motor neurons release this toxic factor into the extracellular microenvironment from where it is taken up by neighbouring cells. Thus cell death may spread in a propagative manner from the onset site. A toxic factor has not been identified, but it has been reported that SOD1 is secreted to the extracellular matrix [13], [14] and thus secreted, misfolded forms of SOD1 could be the toxic species responsible for the transmission of neuronal death, if motor neurons were particularly sensitive to mutant SOD1.

Pathogenic variants of SOD1 exert toxicity by gaining a new biological function. A current view is that this novel function involves an increase in the propensity of SOD1 to oligomerize with itself or with other proteins and thereby to form some type of aggregated species [15], [16], [17], [18]. It is possible that different SOD1 mutants have varying tendencies to aggregate [19] and it is the initial nucleation event of aggregation leading to formation of a stable protein ‘seed’ that is the critical point in the misfolding pathway – similar to that proposed for prion propagation [20], [21]. The evidence of SOD1 secretion and the known phagocytic ability of neurons [22] has led us to test the protein aggregation theory within the prion paradigm. To do this, SOD1 fibrillization assays with varying solvent conditions (pH and chaotrope concentration) were undertaken and we showed *in vitro* that misfolding of SOD1 protein leads to the formation of amyloid fibrils which can seed the formation of further fibrils in an autocatalytic cascade. Our results were consistent with similar published fibrilisation experiments [15], [23], [24], [25], [26], [27] and we have gone on to demonstrate the relevance of the seeding phenomenon to *in vivo* disease by seeding fibrillization reactions with spinal cord homogenates from an ALS transgenic mouse model (tgSOD1^G93A^ [28]) that overexpresses a human fALS mutant SOD1. We found the same seeding ability with these homogenates of affected tissue as was seen with fibrils of recombinant protein. This confirms for the first time that in an ALS model, amyloid seeding activity is present in affected tissues.

These findings provide us with a possible disease mechanism of ALS both in familial and sporadic disease: the spread of ALS pathology from an initial focal point can be accounted for by the seeding of amyloid fibrils in a transmissible process analogous to that of prion propagation. Therefore inhibition of SOD1 fibrillisation protentially provides a point for therapeutic intervention that could halt the progression of disease.

## Results

### SOD1 fibrillization can be potentiated over a wide range of destabilizing conditions

**Figure 1:**
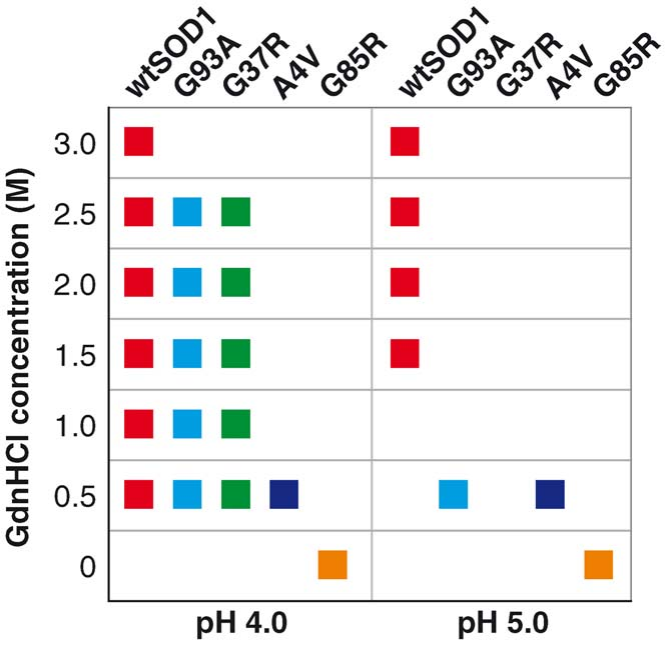
Conditions screened for SOD1 fibrillisation. SOD1 variants with higher stability were able to form fibrils over a wide range of conditions (i.e. number of combinations of pH and denaturant strength) characterized by variation in lag times (wtSOD red square = 10 combinations; G93A light blue square = 6 combinations; G37R green square = 5 combinations; A4V dark blue square = 2 combinations; G85R yellow square = 2 combinations). For the more stable proteins, when incubated in increasing denaturant strength, lag time for fibrils to form is shortened (data not shown).

Prior to characterizing the fibrillization properties of SOD1, all protein preparations were characterized biophysically and biochemically (Supporting Information S1, Figures S1, S2, S3, S4 and Tables S1 and S2) to ensure the SOD1 proteins used in subsequent assays were comparably metallated and had an intact intra-disulfide bond, when validated against other published reports. To screen and select for a condition which would be conducive for SOD1 to form oligomers and amyloid-like fibrils, human SOD1 proteins – wildtype SOD1 (wtSOD1) and four fALS causative mutations, G93A, G37R, A4V, and G85R, were incubated in a matrix of 28 varying solvent conditions (pH and chaotrope/denaturant concentration), totalling 140 screened conditions for all five proteins studied. We monitored fibril formation by following the change in Thioflavin-T (ThT) fluorescence. Figure 1 shows the conditions screened (a gradient of guanidium chloride (GdnHCl) concentration from 0.0 to 3.0M at pH4.0 or 5.0) which gave a positive change in ThT during the assay, represented by color-coded squares for each SOD1 variant. A positive change in ThT was indicative of fibril formation, which was further confirmed by electron microscopy. All SOD1 variants were able to form fibrils in at least one combination of pH – either at pH4.0 and/or 5.0 but not at pH 7.5 or 9.0 – and GdnHCl concentration. Fibril formation was more favourable at pH4.0 in mild denaturing conditions (low GdnHCl concentration) and at pH5.0 in slightly harsher denaturing conditions (higher GdnHCl concentration).

### Kinetics of spontaneous and seeded fibrillization of SOD1

**Figure 2:**
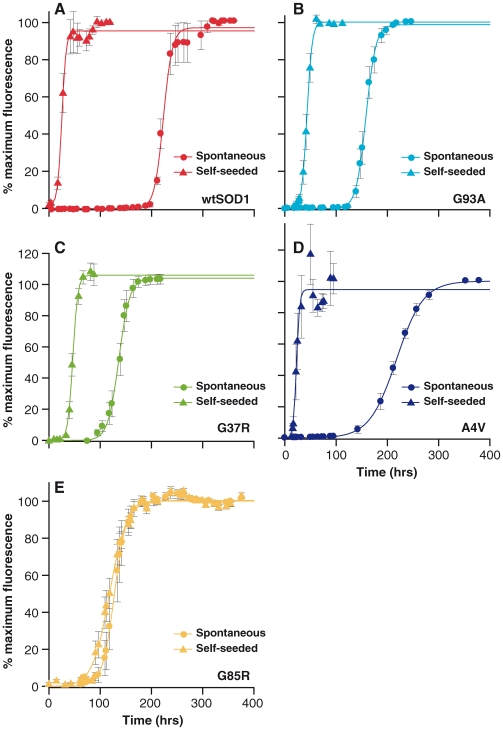
Spontaneous and self-seeded fibrillization of SOD1. Spontaneous and self-seeded reactions for (A) wtSOD1, (B) G93A, (C) G37R, (D) A4V, and (E) G85R. All SOD1 variants, except G85R were incubated at 37°C, at pH4.0 in mild denaturing condition (0.5M GdnHCl). Fibrillization of G85R was performed at pH5.0 with no denaturant. Fibril formation is reported as % maximum ThT relative fluorescence units (RFU) shown as a function of time (hrs). Seeded reactions were spiked with 1% preformed fibrils (v/v). Data shown are average values from 6–10 replicates (± SEM) pooled from 2–3 independent assays. Seeded fibrillization reactions consistently shortened the lag times by an average of 82% (except for G85R) compared to spontaneous reactions under similar conditions.

Spontaneous and seeded reactions were set up to characterise the lag time of fibril formation and additionally for seeded reactions to investigate if addition of preformed SOD1 amyloid fibrils altered the rate of further fibril formation (‘self seeding’, ‘ss’) in an autocatalytic cascade. With the exception of G85R, fibrillization of all SOD1 proteins was carried out at pH4.0 with 0.5M GdnHCl. Fibrillization of G85R was carried out at pH5.0 with no denaturant. The fibrillization lag times of each SOD1 variant were determined for each type of reaction. Figure 2 shows the data fitted fibrillization curve of spontaneous and self-seeded reactions, these values are summarised in Table 1.

**Table 1:** Summary of lag times of SOD1 fibrillization for spontaneous and seeded reactions.

SOD1 variant	F_max_ (a.u.)	T_0.5_ (hrs)	τ (hrs)	Lag time spontaneous	F_max_ (a.u.)	T_0.5_ (hrs)	τ (hrs)	Lag time self-seeded
**wt SOD1**	20618±3517	223.0±0.9	8.7±0.7	205.7±0.5	17073±12201	24.8±0.8	4.4±0.6	16.0±0.6
**G93A**	19041±12443	158.4±0.4	9.6±0.3	139.2±0.3	21964±15221	43.2±0.3	5.0±0.2	33.1±0.2
**G37R**	41649±23461	136.5±0.7	10.7±0.6	115.0±0.4	34286±12334	46.7±0.5	4.9±0.5	37.0±0.4
**A4V**	51191±14760	217.9±0.5	26.2±0.4	165.3±0.4	9080±5952	22.7±1.0	3.1±1.0	16.6±1.0
**G85R**	6507±2199	128.0±0.5	11.8±0.4	104.0±0.3	6992±2205	118.4±0.8	16.7±0.7	85.1±0.5

F_max_ = average maximum fluorescence (arbitrary unit, a.u.), T_0.5_ = observed time at transition mid-point (hrs), lag time = incubation duration prior to increase in fluorescence corresponding to initiation of fibrillization (hrs), τ = 1/propagation rate of fibril growth (hrs). Data shown are average values from 6–10 replicates (± SEM) pooled from 2–3 independent assays.

A comparison of the rate of spontaneous fibrillization showed the lag time for wtSOD1 fibrillization (206 hrs) was considerably longer than for the other SOD1 mutants (mean ± standard deviation = 131±27 hrs). However, in the self-seeded reactions of wtSOD1 the lag time of fibrillization was reduced to 16 hrs, which is consistent with the lag times of self-seeded SOD1 mutants except for G85R (25±11 hrs). The lag time of G85R was excluded from this range because G85R was fibrillized under different conditions compared to other SOD1 variants. Figure 2 shows that while spontaneous reactions (solid circles) had relatively long incubation events, self-seeded reactions (solid triangles) for all SOD1 variants (except G85R) displayed significantly reduced lag times, with an average reduction of 82% (unpaired student t-test, p-value<0.001, when compared to lag times of spontaneous reactions). These findings confirm the generic property of SOD1: both wtSOD1 and mutant SOD1 form fibrils; once formed, these fibrils have the ability to propagate fibrillization in an autocatalytic manner.

**Figure 3:**
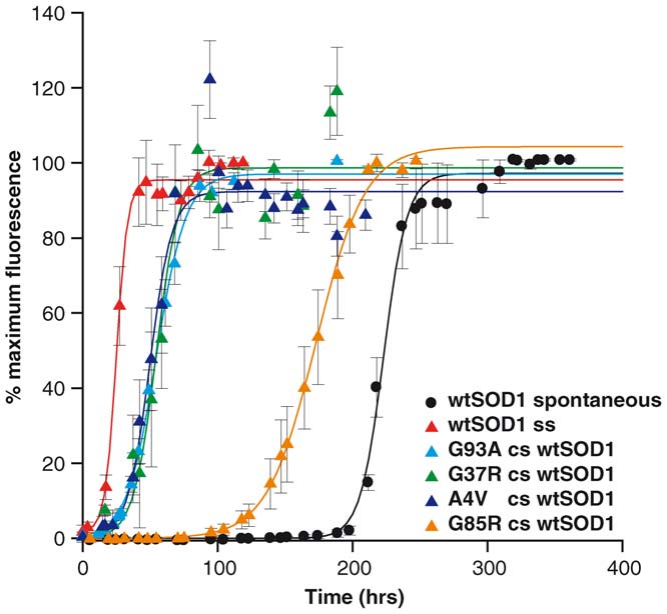
Cross-seeding (cs) fibrillization reactions with preformed fibrils of SOD1 variants into wtSOD1. Formation of fibrils are reported as % maximum ThT relative fluorescence units (RFU) shown as a function of time. Seeded reactions were spiked with 1% preformed fibrils (v/v). Data shown are average values from 6–10 replicates (± SEM) pooled from 2–3 independent assays. Self-seeding of wtSOD1 fibrils into recombinant wtSOD1 (wtSOD1 ss) gave the greatest reduction in lag time with cross-seeding of G93A, G37R, and A4V into wtSOD1 shortening the lag time from 206 hours to between 33 and 38 hours (lag time shortened by approximately 83%). G85R was also capable of reducing the lag time for wtSOD1 fibrillisation but less significantly (33% reduction in lag time).

To investigate if pre-formed fibrils of SOD1 mutants were able to initiate fibrillization of wtSOD1 and reduce the lag time of fibril formation, cross-seeded (‘cs’) reactions were performed for all SOD1 mutants in a similar manner as the self-seeded reactions. All fibrillization reactions were carried out at pH4.0 in the presence of 0.5M GdnHCl. We found reactions cross-seeded with pre-formed fibrils of all SOD1 mutants (except G85R) shortened the lag time of wtSOD1 fibrillization by an average of 83% (from 206 hrs down to 35±3 hrs depending upon the mutation), as shown in Figure 3 and summarised in Table 2.

**Table 2:** Summary of lag times of SOD1 fibrillization for seeded reactions with pre-formed fibrils of SOD1 mutants into wtSOD1 protein and with spinal cord homogenates into G93A and wtSOD1 proteins.

Fibrillisation reaction	F_max_ (a.u.)	T_0.5_ (hrs)	τ (hrs)	Lag time (hrs)
**Cross-seeded (CS) reactions with pre-formed SOD1 mutant fibril (** **Figure 2** **)**				
G93A cs wtSOD1	22214±9547	54.6±0.6	10.9±0.4	32.8±0.3
G37R cs wtSOD1	6482±5934	54.6±2.4	8.5±2.2	37.6±2.0
A4V cs wtSOD1	9828±4252	49.7±2.0	8.3±1.8	33.2±1.7
G85R cs wtSOD1	26714±11056	172.9±1.0	18.1±0.7	136.7±0.5
**Seeded reactions with spinal cord homogenates (** **Figure 4** **)**				
Spinal cord tgSOD1^G93A^ seeded into wtSOD1 protein	2140±690	78.0±0.5	11.0±0.4	56.0±0.3
Spinal cord non- transgenic control seeded into wtSOD1 protein	1352±114	n/a	n/a	n/a
Spinal cord tgSOD1^wtSOD1^ seeded into wtSOD1 protein	1621±210	n/a	n/a	n/a
Spinal cord tgSOD1^G93A^ seeded into G93A protein	7469±4148	34.3±0.8	5.8±0.5	22.6±0.3
Spinal cord non- transgenic control seeded into G93A protein	2702±444	111.1±1.9	15.0±1.4	81.1±0.9
Spinal cord tgSOD1^wtSOD1^ seeded into G93A protein	2077±366	115.6±2.5	14.8±2.1	86.0±1.7

F_max_ = average maximum fluorescence (arbitrary unit, a.u.), T_0.5_ = observed time at transition mid-point (hrs), lag time = incubation duration prior to initiation of fibrillization (hrs), τ = 1/propagation rate of fibril growth (hrs), tgSOD1^G93A^ = transgenic SOD1 mice carrying the G93A mutation, tgSOD1^wtSOD1^ = transgenic wtSOD1 overexpressor mice; n/a = not available. Data shown are average values from 4 to 10 replicates (± SEM) pooled from 2 to 3 independent assays.

#### Testing spinal cord homogenates to determine the in vivo relevance of SOD1 fibrillization

As shown in Figures 2 and 3, SOD1 has a generic property of forming, and seeding formation of, amyloidogenic structures *in vitro*. To start to investigate the *in vivo* relevance of these results, fibrillization assays were undertaken with tissue homogenates from spinal cord of mice, seeded into wtSOD1 and G93A protein preparations. Spinal cord homogenates were taken from three different mouse strains: (1) tgSOD1^G93A^ transgenic mice carrying the pathogenic SOD1 G93A mutation; (2) tgSOD1^wtSOD1^ transgenic mice overexpressing wildtype human SOD1 at a similar level to the tgSOD1^G93A^ mice; these mice do not succumb to motor neuron disease; (3) wildtype control non-transgenic littermates from the tgSOD1^G93A^ colony. Spinal cords were obtained from animals at 120 days old, corresponding to the clinical end-stage age of tgSOD1^G93A^ mice on the genetic background used.

**Figure 4:**
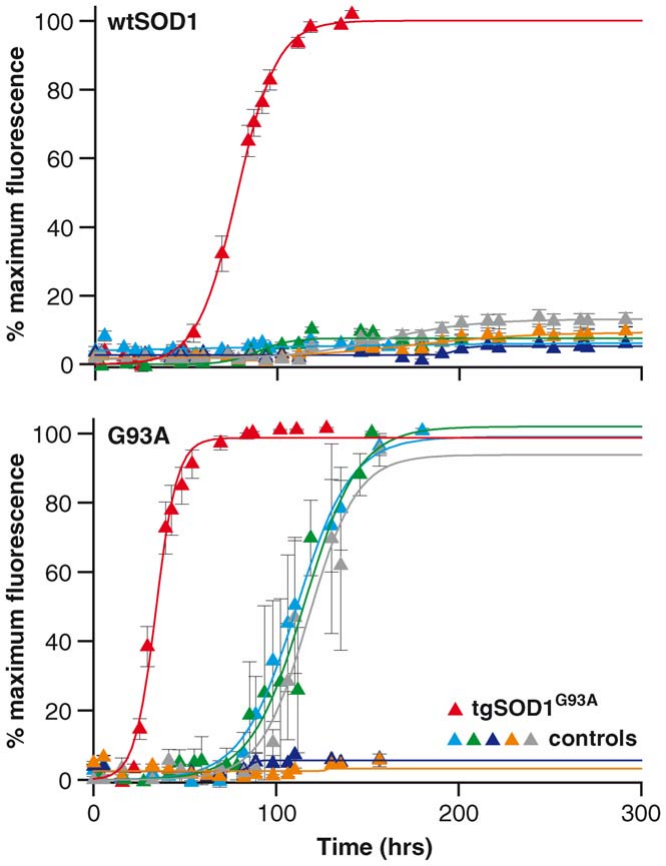
Fibrillogenic seeding with affected tissue homogenates. wtSOD1 (top graph) and G93A (bottom graph) proteins were seeded with spinal cord homogenates from 120 days old tgSOD1^G93A^ (red triangle), tgSOD1^wtSOD1^ (light blue triangle) and control non-transgenic littermates of the tgSOD1^G93A^ (green triangle) mice. Seeded reactions spiked with 1% (v/v) tgSOD1^G93A^ spinal cord homogenates into wt SOD1 and G93A proteins shortened the lag times to approximately 56 hrs and 23hrs respectively, comparable to seeded reactions with pre-formed recombinant G93A fibrils to wt SOD1 and to self-protein (G93A) (refer to Table 1). The lag times of seeded reactions with tgSOD1^wtSOD1^ and non-transgenic control spinal cord homogenates into G93A protein were 1.5–3.5 fold longer than the lag times of seeded reactions by tgSOD1^G93A^ spinal cord homogenates into G93A protein. Seeding with tgSOD1^wtSOD1^ and non-transgenic control spinal cord homogenates into wtSOD1 protein did not give rise to any ThT fluorescence change, indicating an absence of fibril formation. Additional spinal cord homogenate controls were used to test for seeding specificity as described in the text: spinal cord homogenates were prepared from clinical endstage Tg20 mice infected with the RML strain of prions (dark blue triangle), huntingtin transgenic mice (N171-82Q) (grey triangle), and brain homogenate prepared derived from an FTD3 patient (yellow triangle).Data shown are average values from 4–10 replicates (± SEM) pooled from 2–3 independent assays.

Results from these spinal cord homogenate-seeded reactions are shown in Figure 4 and the lag times are summarised in Table 2. In seeding reactions of wtSOD1 and G93A mutant proteins by tgSOD1^G93A^ spinal cord homogenates, fibrillization was initiated at 56 hrs and 23 hrs respectively. This is comparable to the lag times of self-seeded recombinant G93A protein (33 hrs) and cross-seeded recombinant G93A into wtSOD1 protein (33 hrs). Seeded reactions with tgSOD1^wtSOD1^ and non-transgenic control spinal cord homogenates into G93A protein gave lag times of approximately 81 hrs and 86 hrs respectively. Although these numbers appear to be shorter than the lag times for spontaneous reactions (156±39 hrs; G85R data excluded), the difference was determined to be statistically insignificant, using an unpaired student t-test giving a p-value of 0.07. As expected, there was no significant ThT fluorescence change when non-transgenic control spinal cord homogenates were used to seed wtSOD1 protein, indicating no fibrillization. However, we note that SOD1 aggregates have been reported in older wtSOD1 transgenic mice (∼600days) [29], and it would be interesting, for future experiments, to investigate if these aggregates found in older mice have any seeding ability also.

To test for the specificity of the seeding effects found with spinal cord homogenates from end stage tgSOD1^G93A^ mice, spinal cord homogenates of non-ALS related mouse models and brain homogenate from a human neurodegenerative disease were assayed. Spinal cord homogenates from Tg20 mice infected with the RML strain of prions [30], huntingtin transgenic mice (N171-82Q) [31], and human brain homogenate from frozen post mortem material of a patient who died with frontotemporal dementia from a *CHMP2B* mutation (FTD3 [32]) were used as seeds in the same *in vitro* fibrillization assay. These controls showed no significant ThT fluorescence change when seeded into wtSOD1 or G93A proteins (Figure 4). Thus the significantly shortened seeding lag time found with tissue homogenates from tgSOD1^G93A^ mice is not simply associated with a generic process of neurodegenerative disease but rather due to the presence of a specific species of SOD1, found in the spinal cord of tgSOD1^G93A^, which has similar seeding properties to recombinant SOD1 fibrils formed *in vitro*.

### Qualitative characterization of ThT-positive SOD1 species by electron microscopy

**Figure 5:**
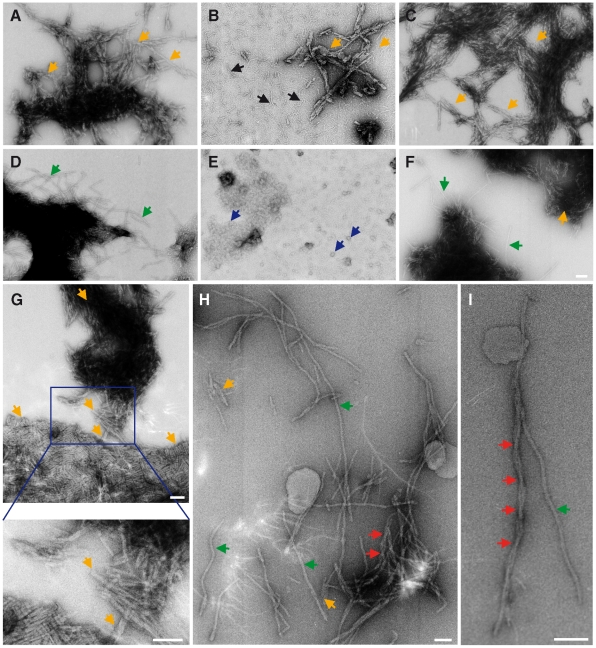
Representative electron micrographs of SOD1 fibrils from spontaneous and seeded reactions (self-seeded and cross-seeded with pre-formed fibril/oligomer or with spinal cord tissue homogenate). Samples from fibrillization reactions of (A) A4V spontaneous, (B) A4V self-seeded, (C) A4V cross-seeded samples into wtSOD1 protein, (D) G37R spontaneous, (E) G85R spontaneous, (F) G85R self-seeded, (G) G93A spontaneous, (H–I) tgSOD1^G93A^ spinal cord homogenate seeded into G93A protein. All spontaneous, self- and cross-seeded reactions for all SOD1 variants were carried out at pH4.0 with 0.5M GdnHCl, except for G85R which was carried out at pH5.0 without any denaturant. Shorter fibrillar fragments are indicated by yellow arrows, which were observed in all samples. Green arrows point to longer and continuous fibrillar threads, whereas black arrows indicate thinner and less regular SOD1 species (possibly shorter oligomers). Blue arrows in (E) point to a less structured morphology of G85R ThT-positive species (most appear as spherical aggregates but some have a toroidal appearance) a morphology which was also rarely observed in other SOD1 fibrillar samples. Red arrows in (H) and (I) indicate flexion points of what appears to be 2–3 protofilaments together to form a thicker fibrillar thread or fragment. All SOD1 fibril samples which were qualitatively characterized under EM showed a common property of increased propensity to clump together or to aggregate, regardless of the morphology of the ThT-positive species. Scale bar = 400nm.

ThT-positive SOD1 samples were examined by electron microscopy (EM) to determine if we could detect species with a fibrillar, amyloid-like morphology. Random ThT-positive SOD1 samples were selected for qualitative EM characterization. Higher order structures resembling amyloid were observed for all samples analysed, which confirmed the change in fluorescence during fibrillization reactions was indeed due to formation of fibrils. These amyloid structures varied in length, Figure 5, from shorter fragments (yellow arrows) to longer thread-like fibrils (green arrows) and in thickness from a thin wiggly appearance (black arrows) to thicker fibrils with 2–3 intertwined protofilaments (red arrows). Occasionally, a less structured morphology was also observed in some ThT-positive SOD1 samples. In Figure 5(E), G85R ThT-positive species mostly appeared as spherical aggregates and some possessed a doughnut-like appearance. The significance of the difference in morphologies is unknown, but the common property observed in all ThT-positive SOD1 samples was the increase in the propensity of the most structurally altered SOD1 mutants to aggregate, apparently independently of the adopted morphology of the ThT-positive species.

### Destabilization of SOD1 is a predisposing property of fibrillization

**Figure 6:**
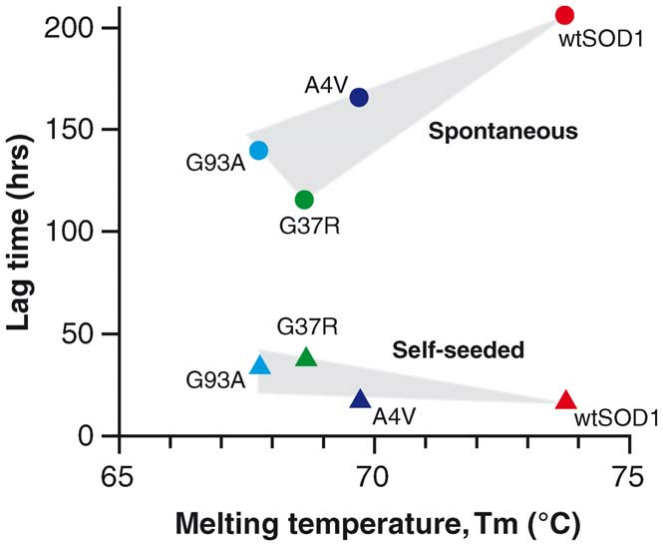
The effect of protein stability on the lag time of SOD1 fibrillization for spontaneous and self-seeded reactions. The plot shows the correlation of SOD1 stability, in the metallated and intra-subunit intact form, (expressed as melting temperature, Tm (°C)) to lag time (hrs). Destabilisation of SOD1 correlated with shorter lag times for fibrillisation in spontaneous reactions, whereas reactions initiated with pre-formed fibrils had lag times that were independent of the original native fold stability. (Tm data for G85R not available, thus it was not possible to include the G85R fibrillization lag time and propagation rate in the graphs.)

By plotting temperature values for the half-point of thermal denaturation (Tm) (Supporting Information S1) against the fibrillization propagation rates and lag times of all SOD1 variants for both spontaneous and seeded reactions (Figure 6), the fibrillization propensity of SOD1 can be correlated with protein stability. In spontaneous fibrillization reactions, SOD1 variants with lower protein stability (reflected by lower Tm values) had a higher propensity to form fibrils quickly (shorter lag time; Figure 6). However, in seeded reactions, there was no appreciable correlation of protein stability to lag time although there was a trend that higher stability may promote seeded fibrillization. This is in line with the aggregation theory proposed by others where destabilization of SOD1 promotes formation of aggregates, which are detrimental to motor neurons [33], [34], [35], [36], [37], [38], [39]. Here, it is demonstrated that formation of fibrils is an alternative misfolding property of destabilized SOD1 resulting in higher order structures with the intrinsic ability to autocatalyze the formation of more fibrils as opposed to the formation of poorly-defined or unstructured aggregates.

## Discussion

The altered biological function that results in mutant SOD1 toxicity and leads to ALS pathology remains obscure. Here, we demonstrate the ability to form self-propagating amyloid structures is a common property of wildtype, G93A, G37R, A4V, and G85R SOD1, and that cross-seeding of pre-formed mutant fibrils, or spinal cord homogenate for tgSOD1^G93A^ animals into wildtype SOD1 can also induce conformational change in wtSOD1 to form amyloid. This property, possibly toxic under certain abnormal cellular microenvironments, is potentially how SOD1 may be led into a propagating misfolding pathway that results in cell death and clinical pathology.

In contrast to earlier reports where SOD1 was used in the demetallated and disulfide reduced state [15], [23], [24], [25], [26], [27], we used the intact and active SOD1 as the starting material in our fibrillization assay (Supporting Information S1).

Both wildtype SOD1 and its mutant variants were able to fibrillize in a variety of conditions and in common with other amyloidogenic proteins, we found that SOD1 variants were able to form fibrils more readily at low pH. This is consistent with previous reports [25], [40], [41] demonstrating the ability of demetallated and reduced forms of SOD1 to form amyloid between pH3.0 and 5.0 with the addition of 0–2M GdnHCl. The similarities of our results with those of others suggest the fibrillizing property of SOD1 does not depend upon aberrant protein modifications, which may occur during protein production and purification.

The spectrum of guanidinium-induced destabilizing conditions at a fixed pH suggests that fibril formation is a conformation-dependent process, with some limited degree of unfolding and destabilization required without complete denaturation to a random coil state in order to expose the critical interaction domains required for oligomerization. SOD1 variants with higher stability were able to form fibrils over a broad range of fibrillization conditions but with a predictable trend whereby denaturing activity was inversely proportional to lag time for fibril formation, This was consistent with the work of Oztug-Durer and colleagues [41] who showed the lag time for fibril formation was shortened with increasing GdnHCl concentration.

This could also explain why G85R, which is markedly destabilised compared to the wildtype protein (Figure S1, Spectroscopic characterisation by far and near UV circular dichroism), failed to form fibrils at pH4, 0.5M GdnHCl, in the presence of any denaturant. It is likely that in the presence of GdnHCl, G85R may undergo excessive unfolding, thus losing the capacity to adopt the correct misfolded conformation needed for oligomerization. Even at pH5.0, G85R is already significantly destabilised which reduces the likelihood of G85R favourably adopting or misfolding to the correct propagating conformation, thus, rendering G85R less responsive, in comparison to other more stable SOD1 variants, to the presence of a template in promoting fibrillar growth.

Seeded reactions clearly demonstrate that SOD1 fibrils possess the ability to seed the formation of further fibrils in an autocatalytic manner and significantly reducing the lag time of fibrillization (in both self-seeded and cross-seeded reactions). Remarkably, a similar seeding effect was observed using tissue homogenates from tgSOD1^G93A^mice (but not from tgSOD1^wtSOD1^), which was capable of stimulating the fibrillization of both wildtype and mutant protein. At first glance, by comparing lag times in seeded reactions, it may appear as if non-transgenic wildtype and tgSOD1^wtSOD1^ spinal cord extracts have seeding activities. However, analysis showed that although the lag times (for G93A seeding reactions using these extracts) appear to be smaller than lag times for recombinant proteins subjected to spontaneous fibrilization reactions under identical fibrillizing condition (i.e. pH4, GdnHCl 0.5M), the difference is statistically insignificant (p = 0.07, calculated using unpaired student t-test). Thus although these extracts may give rise to an apparent slightly shorter lag time, they do not possess a statistically significant seeding activity, thus further supporting our proposal that a specific conformer in the spinal cord extracts of tgSOD1^G93A^ (likely mutant SOD1, in misfolded form/s) may be responsible for the seeding effects observed in the seeding assays. The absence of any seeding activity in tissue homogenates derived from tgSOD1^wtSOD1^ mice adds further credence to the hypothesis that seeding activity is related to ALS pathology as tgSOD1^wtSOD1^ mice do not develop any detectable clinical phenotype, explicable by an absence of amyloid and amyloidogenic seeds within this paradigm.

Potentially, the formation of amyloid fibrils *in vivo* can be induced when the cellular microenviroment is unhealthy and damaging, for example, in conditions of increased oxidative stress. Since tgSOD1^wtSOD1^ mice do not succumb to motor neuron disease, it is likely that the *in vivo* conditions are physiologically healthy, and so the cellular microenviroment is not sufficient to promote the formation of wtSOD1 fibrils in these mice. This is further supported by the other tissue seeding controls used (spinal cord from non-transgenic wildtype littermates, Tg20 mice infected with RML strain of prions, huntingtin transgenic mice, and brain homogenate from an FTD3 patient) in which no seeding effect was observed. Tg20(RML) and huntingtin mice are amyloidogenic models of prion and huntingtin diease, and both mouse strains have been shown to form amyloidogenic proteins in the brain [42], [43], [44]. Here, the absence of a seeding effect when using homogenates from these mouse strains indicates that the SOD1 fibrilisation mechanism is a protein specific process. This is consistent with the active ‘seed’ in the tgSOD1^wtSOD1^ spinal cord homogenate being a conformer of SOD1.

A limitation of this study lies in the definitive identification of SOD1 fibrils as the propagative-SOD1 conformer or ‘template’ in these homogenates responsible for the seeding effects. Although we did not demonstrate the presence of SOD1 fibrils in the spinal cord homogenates we used, others have shown that amyloid fibrils and Thioflavin-reactive inclusions are present in the spinal cord of tgSOD1^G93A^ mice and mouse models expressing G37R and G85R [45]. Additionally, histopathological analysis of spinal cord sections demonstrated via electron microscopy, that these fibrillar structures were immunopositive for human SOD1 [46], [47]. Thus given these results on the presence of SOD1-positive fibrillar aggregates in the spinal cord of SOD1-ALS mouse models, it is reasonable to speculate that the ‘seed’ responsible for seeding may be SOD1 pre-fibrillar aggregates present in the sample or produced from partial dissociation of the monomers from fibrils [48]. However, we note that it remains a possibility that diseased spinal cord contains other factors that may have some effect on seeding. If this is true, this could explain why the kinetics of fibrillization using homogenates *versus* purely recombinant ‘seed’ is slightly different, although not statistically significant.

The present findings parallel clinical observations of disease progression in humans and in mouse models. Classical human ALS cases display a long latent pre-clinical phase with disease onset in mid-life, and usually a rapid disease progression with death occurring within 2–5 years; a phenotype mirrored in ALS mouse models. The late onset of disease is analogous to the long lag time observed in spontaneous fibrillization reactions suggesting nucleation and the establishment of fibril seeds is the rate-limiting step in pathogenesis. Furthermore, the aggression of the disease upon onset is consistent with the *in vitro* fibrillization profile, where by once fibrils are formed further recruitment and oligomerization of SOD1 is a rapid process limited only by substrate concentration. The cell type specificity of this disorder remains a mystery, but may be associated with a sensitivity of these cells to SOD1 aggregates, and perhaps to the phagocytic capacity of motor neurons which promotes uptake of mutant SOD1 proteins [22].

Given the ability of SOD1 to be secreted from cells [13], [49], [50], [14], [51], non-cell autonomous toxicity of mutant SOD1 [52], [53], [54], the acquisition of mutant properties by wtSOD1 when oxidized or misfolded [55] and the clinical observation of disease focality, spread of paralysis and the gradient of motor neuron loss in the spinal cord from onset site [11], [12], we propose a mechanism of disease pathogenesis arising from the generic property of SOD1 to form amyloid. We suggest that in certain cellular and possibly also extra cellular conditions, the highly abundant protein SOD1 can form stable amyloid fibrils associated with self-propagating amyloidogenic seeds. Amyloid fibril formation is promoted significantly by mutations, but is also a property of the wildtype SOD1 protein. The amyloidogenic nuclei of SOD1 may be transferred from one cell to another in a propagative manner in line with the non-cell autonomous theory involving motor neurons, microglia and astrocytes. We hypothesize that toxic SOD1 conformers are formed as part of this replication process analogous to the proposed formation of toxic species in other protein misfolding disorders such as prion disease [20], [21] and tauopathies [56].

We propose that in many ways SOD1 follows a “prion-like” mechanism by seeding the transformation of native SOD1 into fibrillar species which may transmit this transforming ability from cell to cell. Fibrillar or pre-fibrillar species are presumed to be toxic the spread of which would result in a progressive loss of cells from the site of onset. We have chosen to use the term “prion-like” because of the nucleation dependent polymerization property of SOD1, as observed in the seeding experiments. This seeding effect was not confined to seeding into an identical primary sequence, but can also cross-seed between minor mismatches in sequence from mutant to wild-type proteins sharing some analogy to human prion disease. Although our data is limited to the propagation of altered protein confromation and we have not explored the potential cytotoxicity of fibrillar and pre-fibrillar SOD1 these could be the transmissible factors resulting in spread of motor neuron cell death.

The impact of this proposed mechanism is exciting because it points towards the possibility of a practical approach to the treatment of ALS, both familial and sporadic cases. Slowing down or even halting disease progression after onset could be achieved by identifying compounds that inhibit amyloid formation either by stabilizing the native conformation of SOD1 or by directly inhibiting the fibrillization process, as proposed in prion disease [57]. Indeed suggestions from other neurodegenerative diseases indicate that minor reductions in the propagation rates of toxic assemblies can allow normal cellular clearance mechanism to degrade amyloidogenic nuclei thereby providing a potential treatment and perhaps ultimately a cure.

## Materials and Methods

### Ethics statement

All work involving mice was carried out under Licence from and in accordance with UK Home Office requirements. This study was approved by the local ethical review panel (ERP) of the MRC Prion Unit.

### Expression, purification and characterization of human SOD1 variants

SOD1 proteins, wildtype and four mutants (G93A, G37R, A4V and G85R), were produced in and purified from an *E.coli* expression system. All expression constructs were based on the cSOD1 plasmid [58], which had been modified as described and subcloned into the pET28 expression vector (Novagen) [59]. Each protein preparation was subjected to characterization of their functional activity using a commercial assay kit (Oxis), secondary and tertiary structure assessment using circular dichroism (CD), protein thermo-stability evaluation using differential scanning fluorimetry (DSF) and SOD1 dimerization status determination using analytical ultracentrifugation (AUC) (see Supporting Information S1).

### Preparation of tissue homogenates

Spinal cord obtained from sacrificed mice were homogenised as a 10% w/v preparation in Dulbecco's phosphate-buffered saline (PBS) lacking Ca^2+^ and Mg^2+^ (Sigma) using a Duall tissue grinder (Anachem).

### In vitro conversion of SOD1 into amyloid fibrils

To form amyloid fibrils a stock solution of SOD1 protein, wildtype and mutants (G93A, G37R, A4V, G85R) (1.3–1.5 mg/ml) was diluted to a final protein concentration of 10µM in 20mM Tris-acetate buffer, with varying concentrations of guanidium hydrochloride (GdnHCl) and at varying pH (4.0, 5.0, 7.5 and/or 9.0), containing a final concentration of 10µM Thioflavin-T (ThT). Four Hybaid Ribolyser beads were placed into each well of a 96-well transparent flat-bottom plate; then 200µl reaction mixture containing diluted protein and ThT was pipetted into wells, and the plates were covered using an optically corrected adhesive film. The plates were incubated at 37°C with continuous shaking at 830 rpm using a plate incubator shaker (GrantBio). The kinetics of fibril formation were monitored by taking time point measurements of fluorescence emission at 485nm when excited at 450nm using a fluorimeter plate reader (Spectrofluor, Tecan). For seeded reactions, 2µl of preformed fibrils from a previous spontaneous reaction or 10% w/v tissue homogenate were added to 200µl reaction as described above.

The lag phase of amyloid formation was determined by fitting the time-dependent changes in the fluorescence of ThT (*F*) overtime of the reaction (*t*) to the following equation:
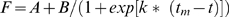
where *A* is the base level of ThT fluorescence during the lag phase, *B* is the difference between final level of ThT fluorescence at plateau and the initial level during the lag phase, *k* is the rate constant of fibril growth (h^−1^), and *t_m_* is the observed time at midpoint of transition. The lag time (*t_l_*) of fibril formation was calculated as: *t_l_* = *t_m_*−2/*k* as previously described by Nielsen et al [60].

### Characterization of ThT-positive species by electron microscopy

For negative stain EM, 3.5µl fibril preparation was applied to a carbon-coated, glow-discharged, 300-mesh copper grid and blotted after 2–3 min. The grids were stained with 3.5µl 2% (w/v) uranyl acetate, blotted after 3 min and allowed to air-dry. Images were recorded using minimal electron dose at a magnification of 27,000× in a Tecnai T10 microscope (FEI, Eindhoven, NL) with a tungsten filament operating at 100 kV. The defocus for these images ranged between 300 nm and 1000 nm, allowing the inclusion of data to a spatial frequency of (20Å^−1^) without CTF correction.

## Supporting Information

Supporting information S1 Supporting information: Results and Discussion, Materials and Methods, References.
(0.11 MB DOC)

Figure S1 Spectroscopic characterisation by far and near UV circular dichroism (CD). All proteins displayed spectra which were similar to wtSOD1 with the exception of G85R. Deviation of far-UV CD spectra for G85R from wtSOD1 reflects some degree of loss of secondary structure, but little change in near-UV spectra (inset) reflecting no significant perturbation of aromatic residue environments.
(0.65 MB TIF)

Figure S2 Coomassie Blue stained SDS-PAGE showing the purity of recombinant SOD1 proteins. Positions of molecular weight markers are indicated in kiloDaltons.
(0.58 MB TIF)

Figure S3 SOD1 assembly state as determined by analytical ultracentrifugation (AUC). Sedimentation velocity (SV) profile of SOD1 proteins (at three concentrations) based on the continuous size-distribution (c(S)) model. The analysis included all 200 scans acquired during the experimental runs, which were analysed using the Sedfit software (version 9.2) based on the continuous size distribution model. The larger the s-values, the larger the size of the sedimenting protein species. SOD1 dimers and monomers sedimented between 2.8 and 3.1S and 1.4 and 1.8S, respectively. All proteins except A4V, and G85R existed almost exclusively as SOD1 dimers. A4V and G85R showed a higher propensity to monomerize. G85R had multiple peaks with s-values larger than s-values for dimeric SOD1, indicating that the destabilised G85R has a high propensity to aggregate.
(1.02 MB TIF)

Figure S4 SOD1 thermo-stability as determined using differential scanning fluorimetry (DSF). Protein unfolding was monitored by slow thermal unfolding using the SYPRO Orange reporter dye over a temperature range of 40 to 95°C for: (A) wtSOD1, (B) G93A, (C) G37R, (D) A4V. Protein stability was dependent on both concentration (11uM vs. 33uM) and degree of metallation. Proteins were most stable at higher concentrations, and when metallated. Demetallation with EDTA markedly reduced protein stability at all concentrations analysed by approximately 20°C. It was not possible to plot a thermal denaturation profile for G85R using DSF as G85R was relatively unfolded to begin with, thus giving very high initial fluorescence which makes data plot invalid as no co-operative unfolding transition could be observed.
(0.94 MB TIF)

Table S1 Functional activity of SOD1 variants.
(0.01 MB DOCX)

Table S2 Summary of SOD1 stability profiles.
(0.01 MB DOCX)
