# Supplementary material for: Superoxide Dismutase 1 and tgSOD1G93A Mouse Spinal Cord Seed Fibrils, Suggesting a Propagative Cell Death Mechanism in Amyotrophic Lateral Sclerosis
Source: PLoS One. 2010 May 13;5(5):e10627. doi: 10.1371/journal.pone.0010627 (PMC2869360; doi:10.1371/journal.pone.0010627)
Supplement: Supporting information S1 — Supporting information: Results and Discussion, Materials and Methods, References. (0.11 MB DOC) [file pone.0010627.s001.doc]

Superoxide dismutase 1 and tgSOD1^G93A^ mouse spinal cord seed fibrils, suggesting a propagative cell death mechanism in amyotrophic lateral sclerosis

Ruth Chia^1^, M. Howard Tattum^2^, Samantha Jones^2^, John Collinge^1, 2^, Elizabeth M.C. Fisher^1^,* and Graham S. Jackson^2^

^1^Department of Neurodegenerative Disease, ^2^MRC Prion Unit, UCL Institute of Neurology, London, UK

**Supplementary Information**

**Supplementary Results and Discussion**

Characterization of SOD1 protein preparations

All expression constructs were based on the cSOD1 plasmid [1], which had been modified as described [2]. All purified SOD1 variants were characterized for their biophysical and biochemical properties and were found to be comparable to published data for these variants [3, 4, 5, 6, 7, 8, 9]. The purity of recombinant protein preparations was greater than 95% as judged by SDS-PAGE (Figure S2 Coomassie Blue stained SDS-PAGE showing the purity of recombinant SOD1 proteins) and enzymatic activity was as expected (Table S1, Functional activity of SOD1 variants) indicating proteins were correctly metallated, except where mutations were known to disrupt metal coordination. Similarly secondary and tertiary structural organisation as judged by CD (Figure S1, Spectroscopic characterisation by far and near UV circular dichroism (CD)) was comparable for mutants and wildtype SOD1 with the exception of G85R, which displayed significant perturbation to the native structure. The stability of the native dimer was assessed by analytical ultracentrifugation which revealed G93A and G37R almost exclusively populated the native dimer as did the wildtype protein (Figure S3, SOD1 assembly state as determined by analytical ultracentrifugation (AUC)). The A4V mutation resulted in a slight perturbation of dimer stability with a proportion of inactive monomer being detectable, consistent with the reduced specific activity recorded in Table S1 (Functional activity of SOD1 variants). G85R was found to display a profoundly altered profile with not only a significant quantity of monomer present but also higher order oligomeric and aggregated species. Again this was consistent with observed loss of secondary structure in Figure S1 (Spectroscopic characterisation by far and near UV circular dichroism (CD)) and the dramatically reduced specific activity (Table S1 (Functional activity of SOD1 variants)). The thermal stability of the proteins was investigated by differential scanning fluorimetry (Figure S4, SOD1 thermo-stability as determined using differential scanning fluorimetry (DSF); summarized in Table S2, Summary of SOD1 stability profiles) which further reinforced the near-native character of G93A and G37R mutations. A4V displayed a greater change in T_m_ between metallated and demetallated isoforms which was again consistent with observations of lower specific activity (Table S1 (Functional activity of SOD1 variants)) and a proportion of molecules populating monomeric rather than dimeric states (Figure S3, SOD1 assembly state as determined by analytical ultracentrifugation (AUC)). The disulfide/thiol state of SOD1 was also assayed using Ellmans reagent, and all SOD1 proteins preparations have an intact intra-subunit disulfide bond (data not shown).

Although the degree of metallation of SOD1 was not determined quantitatively, it was possible to make qualitative deductions on the metallation state based on a combination of measured biophysical and biochemical properties of each protein, as mentioned above, and comparing them to known properties published by others. Previously published data by others showed reliable correlations of metallation status and disulfide/thiol status with the selected parameters [3, 10]. We showed that when wtSOD1 was metallated with at least one Zn^2+^/monomer and had an intact intra-subunit disulfide bond, its stability was increased dramatically (melting temperature, Tm, of ~ 75°C, and it existed primarily as dimers, as shown by analytical ultracentrifugation with a single peak at the calculated dimeric molecular weight of ~32kDa.

The metallation of wtSOD1 with Cu was shown by a fully functional active site with a measured activity of 3000 – 4000U/mg, which was reported to contain approximately 1.8mol Cu/mol dimer [11, 12, 13]. Our data showed that wtSOD1 had comparable stability (Tm ~76°C), SOD1 activity (3800U/mg), existed predominantly as dimers and had an intact intra-subunit disulfide bond. These results indicate that wtSOD1 -- and this can likely be extrapolated to other SOD1 mutants tested here, because they behaved similarly to wtSOD1 but with a slight decrease in Tm and lower functional activity due to the presence of mutation -- was likely to also have a metallation degree of Cu and Zn that was comparable to published data, of at least 1 mol Cu and 1 mol Zn/mol monomer of SOD1.

Overall our results suggests that the SOD1 proteins, purified here using an *Escherichia coli* system, used for subsequent characterization are similar to SOD1 proteins expressed in a eukaryotic system. This argues against the possibility that the fibrillization properties observed here are due to conformational differences arising from expressing SOD1 in an *E.coli* system which lacks the machinery for post-translational modification of SOD1.

**Supplementary Materials and Methods**

**Expression and Purification of Human SOD1 Variants**

The sequence of the wildtype human SOD1 cDNA was confirmed by sequencing a preparation from the plasmid stock, pcSOD1, which contains a human SOD1 cDNA cloned into the vector pSP64 and comparing the results to the published cDNA sequence Accession no.: [NM_000454](http://www.ncbi.nlm.nih.gov/entrez/viewer.fcgi?db=nucleotide&val=48762945), from the Nucleotide database in NCBI ([http://www.ncbi.nlm.nih.gov](http://www.ncbi.nlm.nih.gov/)). Using this as template, site directed mutagenesis was performed to generate constructs for four other mutants, here described as G93A, G37R, A4V and G85R. Constructs were cloned into the pET28 expression vector (Novagen), downstream of the T7 promoter between the *NdeI* and *XhoI* restriction sites introducing a thrombin cleavage site between the protein and the N-terminally added His-tag. All constructs were transformed into *E. coli* strain BL21 (DE3) (Novagen) according to the manufacturer’s protocol. SOD1 expression was induced with 0.1M IPTG at 18°C overnight. Recombinant proteins were purified to homogeneity using metal chelate affinity (Ni-NTA, Qiagen) chromatography and the polyHis-tag cleaved with thrombin (Novagen). All Ni-NTA purification was performed at room temperature using a Bio-Rad Bio-Logic chromatography system. Purified SOD1 was metal loaded by dialyzing it against 100mM Tris-Cl pH8.0, 300mM NaCl containing 150-200µM CuSO_4_ over 3-4 hours at room temperature, then followed by ZnSO_4_ and final dialysis into 20mM Tris-Cl pH7.5 for storage.

**Spectroscopic measurements – Circular Dichroism at far and near UV**

Circular dichroism (CD) spectroscopy was performed using a Jasco J-715 spectrophotometer (Jasco). The secondary and tertiary structure of purified, metal loaded SOD1 was determined by obtaining CD spectra at far-UV (250-190nm) and near-UV (350-250nm) respectively. Far-UV CD spectra were measured in a 0.1mm path length circular cuvette, and near-UV in 10mm path length standard quartz cuvette. All data were collected using a stop resolution of 1nm, a scan speed of 50nm/min, and a 1 second response time. Measurements were performed over 10 accumulations to increase signal to noise ratio and baseline corrected against storage buffer. Protein concentrations were accurately determined and varied between approximately 1.0-1.5mg/ml. CD measurements were converted to units of molar ellipticity ([θ]). All corrections and processing were done using the Jasco Standard Analysis Program.

**Measurement of SOD1 Enzymatic Activity**

SOD1 activity was determined at relevant steps throughput purification using a commercially available kit, Bioxytech SOD-525 assay (Oxis Research). Activities for SOD1 variants are expressed as % activity of wildtype SOD1 (wtSOD1).

**Differential Scanning Fluorimetry (DSF)**

Thermal unfolding of SOD1 variants was monitored using SYPRO Orange and was performed using a real-time PCR instrument (Applied Biosystem TaqMan RT-PCR). Experimental design was adapted from published protocol of [14]. Briefly, using a 96-well PCR plate, SYPRO Orange was mixed with protein in 1:1000 ratio with final volume of 20µl/well (triplicates/sample and with/without EDTA at 11µM and 33µM) and plates were sealed with an Optical PCR seal. The RT-PCR instrument was programmed to run with an increasing temperature gradient from 19°C-95°C with 1°C increments and to hold for 60sec at every new temperature. Fluorescence was monitored using the in-built filters: FAM (492 nm) and ROX (610 nm), for excitation and emission, respectively. The fluorescence intensity was plotted as a function of temperature and curve-fitted according to the Vant Hoff equation to determine the Tm value (temperature at the mid-point of unfolding) using GraFit (Erithacus Software Ltd).

**Analytical Ultracentrifugation (AUC)**

Dimer stability of SOD1 variants was determined by sedimentation velocity measures for wtSOD, G93A, G37R, A4V, and G85R in 20mM Tris-Cl, pH7.5, using a Beckman Optima XL-I. 400µl protein was loaded into a double sector Epon cell with sapphire windows and centrifuged at 50,000 rpm at 20°C using an An-50Ti rotor. Samples were monitored immediately on reaching maximum speed using interference optics and then a further 199 scans were taken every 5 minutes. Alternate scans were analysed using Sedfit Version 9.2 using continuous size or c(s) distribution method entering no prior knowledge. Values for the partial specific volume of the protein and density and viscosity of the buffer used, were calculated using Sednterp [15] and entered into the model parameters. This gave a distribution in sedimentation coefficients which was then converted to a molar mass distribution using the c(M) distribution model. c(S) units are fringes per Svedberg, c(M) units are fringes/Da. 1 fringe is approx 0.3mg/ml

References

1. Lieman-Hurwitz J, Dafni N, Lavie V, Groner Y (1982) Human cytoplasmic superoxide dismutase cDNA clone: a probe for studying the molecular biology of Down syndrome. Proc Natl Acad Sci U S A 79: 2808-2811.

2. Stevens JC, Chia R, Hendriks WT, Bros-Facer V, van Minnen J et al. (2010) Modification of superoxide dismutase 1 (SOD1) properties by a GFP tag--implications for research into amyotrophic lateral sclerosis (ALS) PLoS ONE 5: e9541

3. Doucette PA, Whitson LJ, Cao X, Schirf V, Demeler B et al. (2004) Dissociation of human copper-zinc superoxide dismutase dimers using chaotrope and reductant. Insights into the molecular basis for dimer stability. J Biol Chem 279: 54558-54566.

4. Hough MA, Grossmann JG, Antonyuk SV, Strange RW, Doucette PA et al. (2004) Dimer destabilization in superoxide dismutase may result in disease-causing properties: structures of motor neuron disease mutants. Proc Natl Acad Sci U S A 101: 5976-5981.

5. Hart PJ (2006) Pathogenic superoxide dismutase structure, folding, aggregation and turnover. Curr Opin Chem Biol 10: 131-138.

6. Strange RW, Antonyuk S, Hough MA, Doucette PA, Rodriguez JA et al. (2003) The structure of holo and metal-deficient wild-type human Cu, Zn superoxide dismutase and its relevance to familial amyotrophic lateral sclerosis. J Mol Biol 328: 877-891.

7. Strange RW, Antonyuk SV, Hough MA, Doucette PA, Valentine JS et al. (2006) Variable metallation of human superoxide dismutase: atomic resolution crystal structures of Cu-Zn, Zn-Zn and as-isolated wild-type enzymes. J Mol Biol 356: 1152-1162.

8. Valentine JS, Hart PJ (2003) Misfolded CuZnSOD and amyotrophic lateral sclerosis. Proc Natl Acad Sci U S A 100: 3617-3622.

9. Wang J, Caruano-Yzermans A, Rodriguez A, Scheurmann JP, Slunt HH et al. (2007) Disease-associated mutations at copper ligand histidine residues of superoxide dismutase 1 diminish the binding of copper and compromise dimer stability. J Biol Chem 282: 345-352.

10. Furukawa Y, O'Halloran TV (2005) Amyotrophic lateral sclerosis mutations have the greatest destabilizing effect on the apo- and reduced form of SOD1, leading to unfolding and oxidative aggregation. J Biol Chem 280: 17266-17274.

11. Kajihara J, Enomoto M, Katoh K, Mitsuta K, Kohno M (1988) Relation between ESR-detectable Cu(II) and superoxide dismutase activity. J Biochem 104: 855-857.

12. Kajihara J, Enomoto M, Nishijima K, Yabuuchi M, Katoh K (1988) Comparison of properties between human recombinant and placental copper-zinc SOD. J Biochem 104: 851-854.

13. Kang HJ, Choi BJ, Kim SM (1997) Expression and Characterization of Recombinant Human Cu,Zn-Superoxide Dismutase *in Escherichia coli.* Biochem Mol Bio 30: 6

14. Niesen FH, Berglund H, Vedadi M (2007) The use of differential scanning fluorimetry to detect ligand interactions that promote protein stability. Nat Protoc 2: 2212-2221.

15. Hayes DB, Laue TM, Philo J (2003) SEDNTERP (Sedimentiation Utility Software). Amgen Corp
