# Supplementary figures and images for: Superoxide Dismutase 1 and tgSOD1G93A Mouse Spinal Cord Seed Fibrils, Suggesting a Propagative Cell Death Mechanism in Amyotrophic Lateral Sclerosis
Source: PLoS One. 2010 May 13;5(5):e10627. doi: 10.1371/journal.pone.0010627 (PMC2869360; doi:10.1371/journal.pone.0010627)

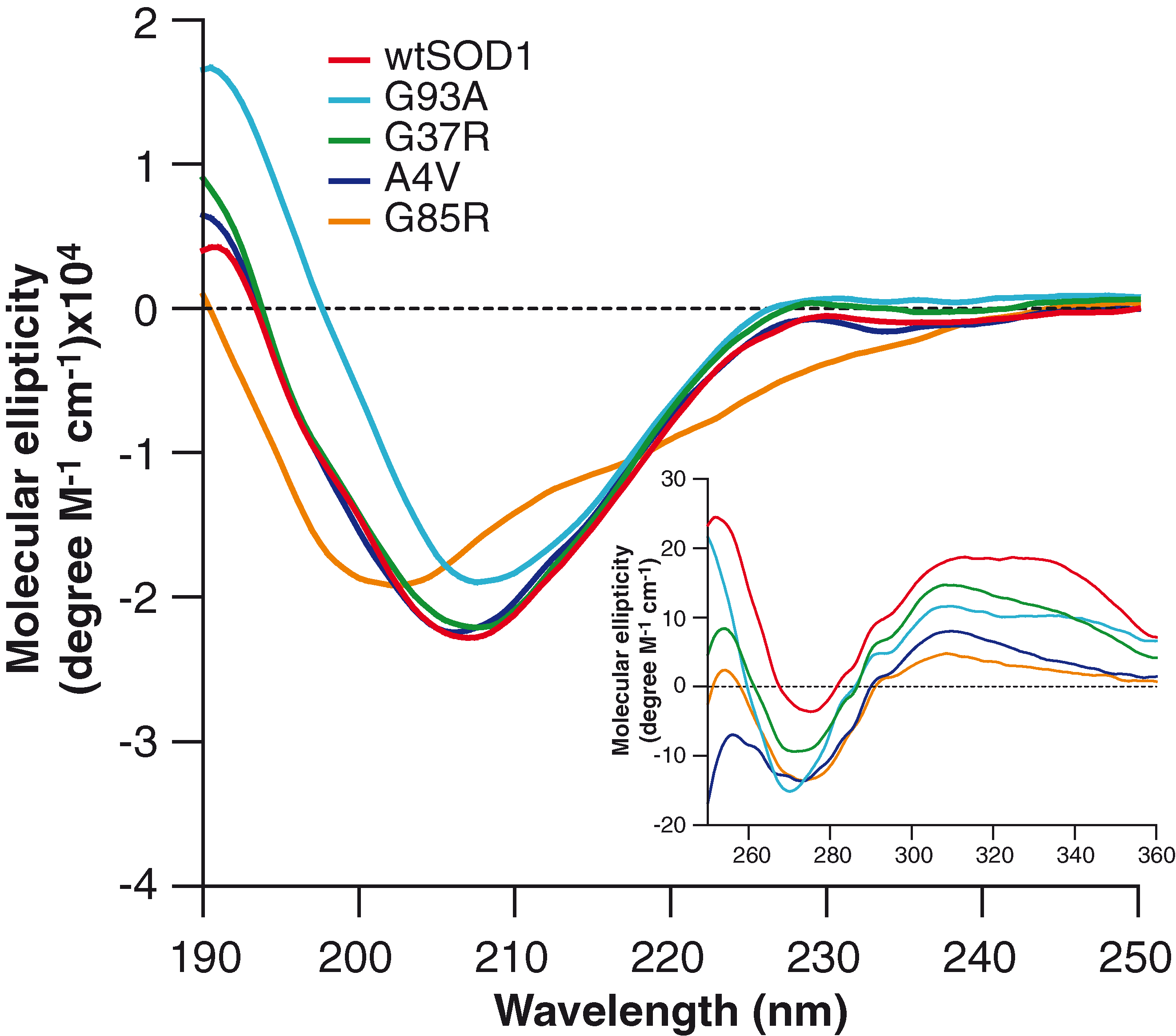

Supplement: Figure S1 — Spectroscopic characterisation by far and near UV circular dichroism (CD). All proteins displayed spectra which were similar to wtSOD1 with the exception of G85R. Deviation of far-UV CD spectra for G85R from wtSOD1 reflects some degree of loss of secondary structure, but little change in near-UV spectra (inset) reflecting no significant perturbation of aromatic residue environments. (0.65 MB TIF) [file pone.0010627.s002.tif]

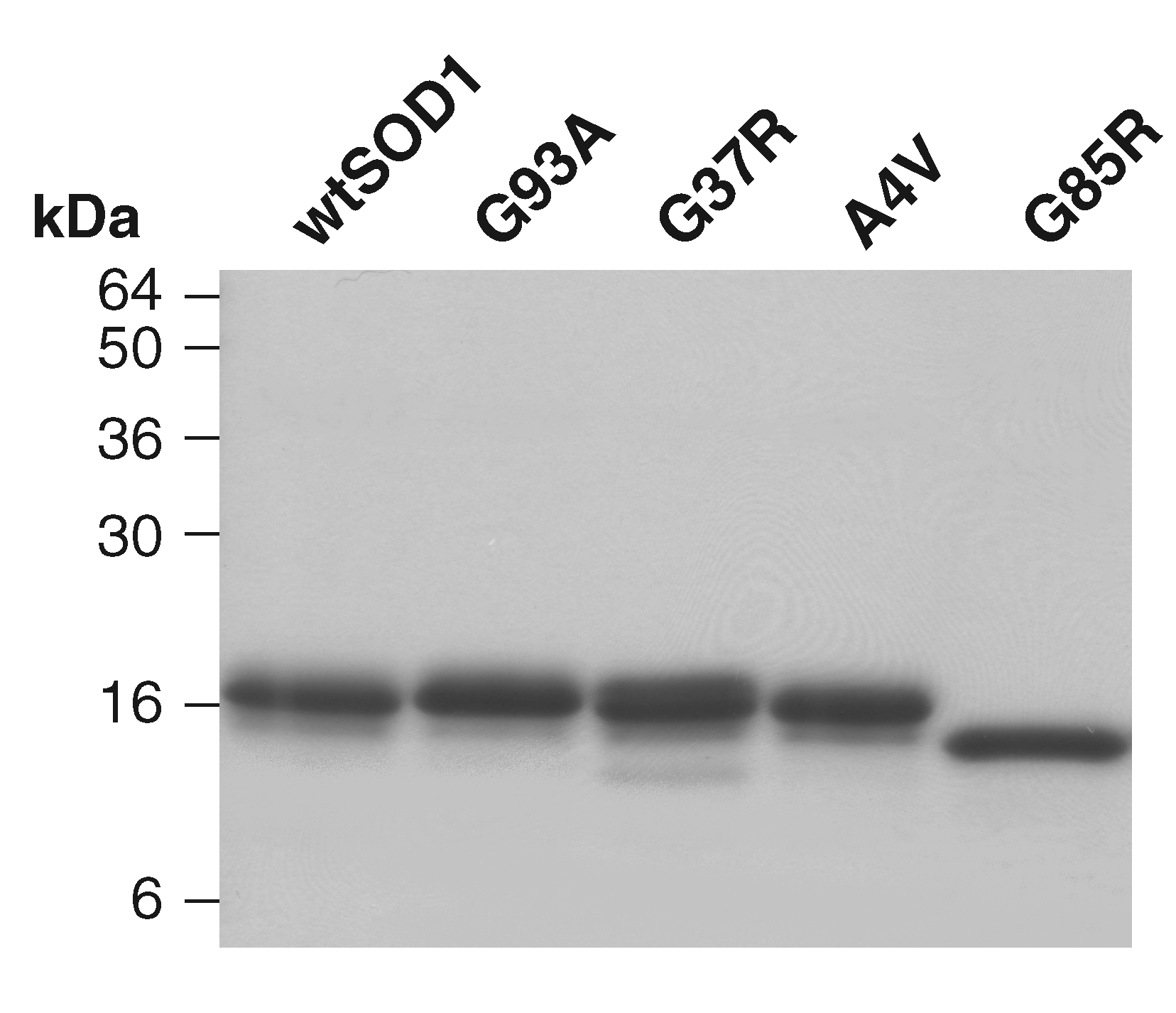

Supplement: Figure S2 — Coomassie Blue stained SDS-PAGE showing the purity of recombinant SOD1 proteins. Positions of molecular weight markers are indicated in kiloDaltons. (0.58 MB TIF) [file pone.0010627.s003.tif]

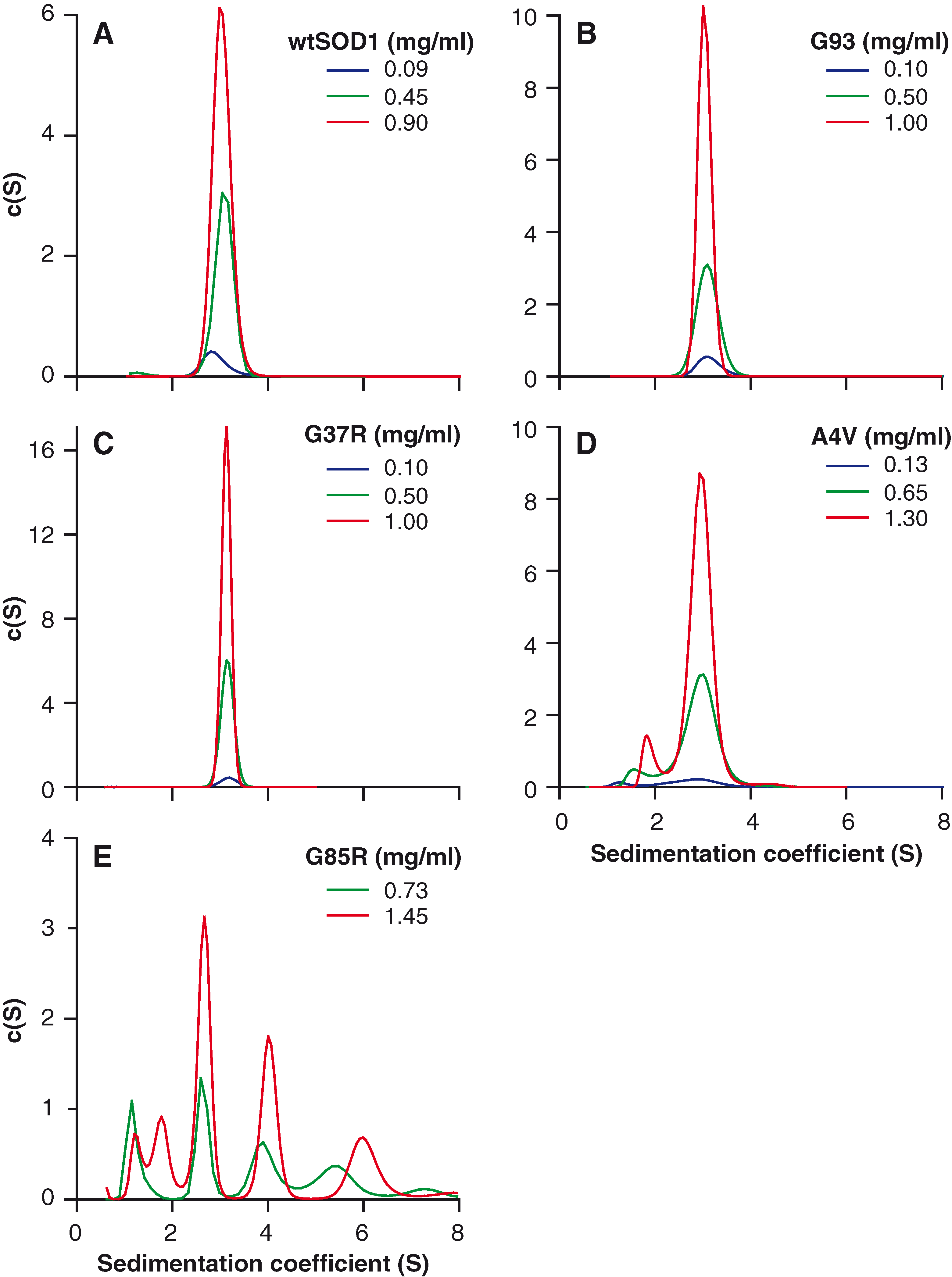

Supplement: Figure S3 — SOD1 assembly state as determined by analytical ultracentrifugation (AUC). Sedimentation velocity (SV) profile of SOD1 proteins (at three concentrations) based on the continuous size-distribution (c(S)) model. The analysis included all 200 scans acquired during the experimental runs, which were analysed using the Sedfit software (version 9.2) based on the continuous size distribution model. The larger the s-values, the larger the size of the sedimenting protein species. SOD1 dimers and monomers sedimented between 2.8 and 3.1S and 1.4 and 1.8S, respectively. All proteins except A4V, and G85R existed almost exclusively as SOD1 dimers. A4V and G85R showed a higher propensity to monomerize. G85R had multiple peaks with s-values larger than s-values for dimeric SOD1, indicating that the destabilised G85R has a high propensity to aggregate. (1.02 MB TIF) [file pone.0010627.s004.tif]

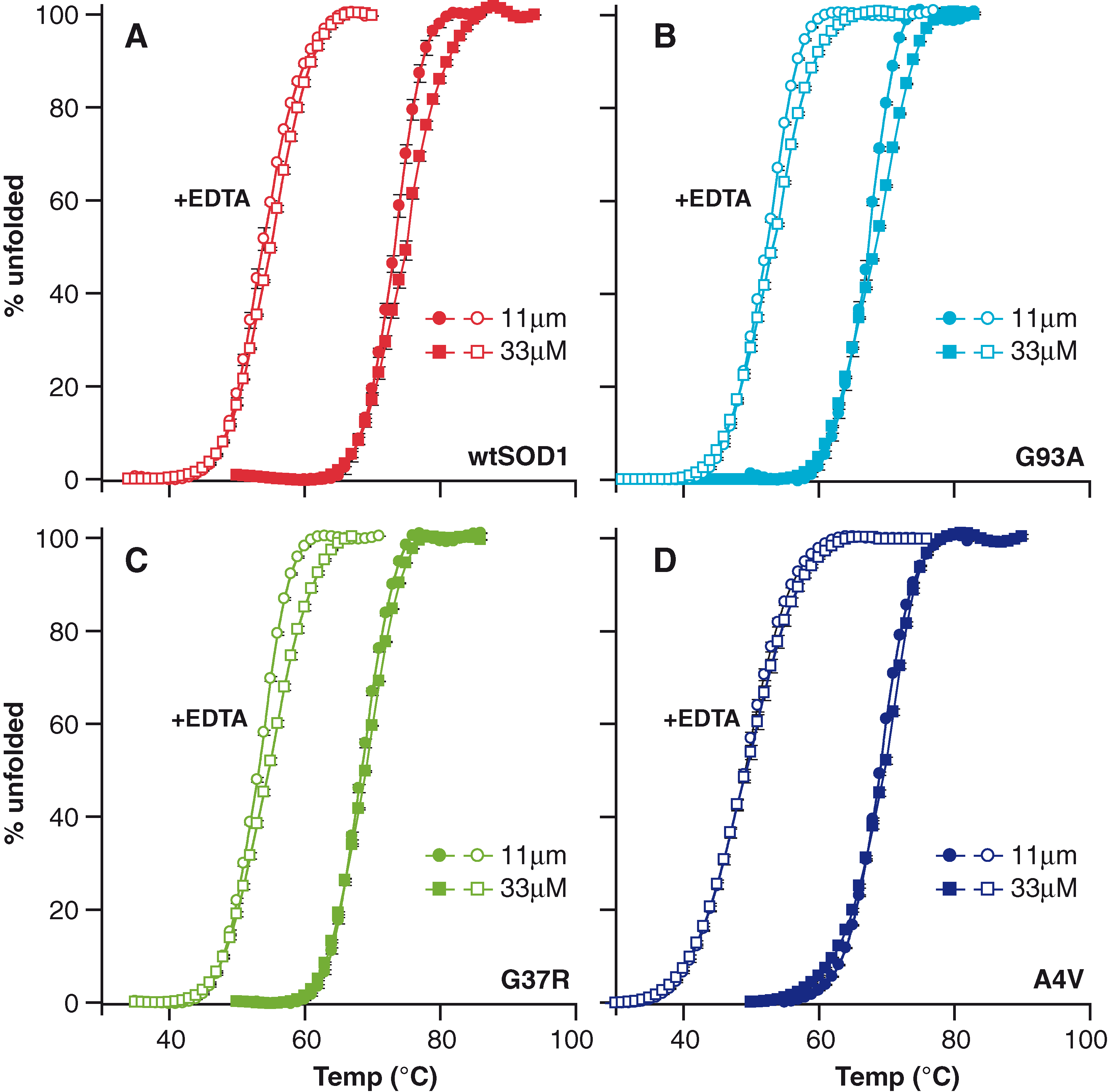

Supplement: Figure S4 — SOD1 thermo-stability as determined using differential scanning fluorimetry (DSF). Protein unfolding was monitored by slow thermal unfolding using the SYPRO Orange reporter dye over a temperature range of 40 to 95°C for: (A) wtSOD1, (B) G93A, (C) G37R, (D) A4V. Protein stability was dependent on both concentration (11uM vs. 33uM) and degree of metallation. Proteins were most stable at higher concentrations, and when metallated. Demetallation with EDTA markedly reduced protein stability at all concentrations analysed by approximately 20°C. It was not possible to plot a thermal denaturation profile for G85R using DSF as G85R was relatively unfolded to begin with, thus giving very high initial fluorescence which makes data plot invalid as no co-operative unfolding transition could be observed. (0.94 MB TIF) [file pone.0010627.s005.tif]
