# Supplementary material for: Superoxide Dismutase 1 and tgSOD1G93A Mouse Spinal Cord Seed Fibrils, Suggesting a Propagative Cell Death Mechanism in Amyotrophic Lateral Sclerosis
Source: PLoS One. 2010 May 13;5(5):e10627. doi: 10.1371/journal.pone.0010627 (PMC2869360; doi:10.1371/journal.pone.0010627)
Supplement: Table S1 — Functional activity of SOD1 variants. (0.01 MB DOCX) [file pone.0010627.s006.docx]

**Table S1. Functional activity of SOD1 variants.**

The enzymatic activity of all proteins was compared to the activity of wtSOD1 and a commercial recombinant SOD1 protein (hu_c_SOD1). Of the mutant SOD1 purified, three have mutations located away from the metal binding/catalytic region, thus having properties similar to wtSOD1. These mutants are denoted wild-type-like SOD1 mutants (WTL) and they are G93A, G37R, and A4V. G85R is a SOD1 variant with mutation within the metal binding/catalytic region (known as metal-binding-region (MBR) mutant) and has a marked reduction in metal binding properties and SOD1 activity compared to wtSOD1. Protein conformations of purified SOD1 (wtSOD, G37R and A4V) were confirmed by crystal structure analysis (data not shown) and were comparable to published data, except for the absence of acetylated N-terminus.

| **SOD1 variant** | **SOD1 specific activity**  **(units/mg)** | **% specific activity of wtSOD1** |
| --- | --- | --- |
| hu_c_SOD1 | 3000 - 4000 | 80-105 |
| wtSOD1 | 3800 | 100 |
| G93A | 3900 | 102 |
| G37R | 2600 | 68 |
| A4V | 1200 | 32 |
| G85R | 290 | 8 |
