# Supplementary material for: Superoxide Dismutase 1 and tgSOD1G93A Mouse Spinal Cord Seed Fibrils, Suggesting a Propagative Cell Death Mechanism in Amyotrophic Lateral Sclerosis
Source: PLoS One. 2010 May 13;5(5):e10627. doi: 10.1371/journal.pone.0010627 (PMC2869360; doi:10.1371/journal.pone.0010627)
Supplement: Table S2 — Summary of SOD1 stability profiles. (0.01 MB DOCX) [file pone.0010627.s007.docx]

**Table S2. Summary of SOD1 stability profiles.**

Tm (^o^C) values are presented as a mean ± SD (n=3).

| **SOD1 variant** | **Apparent Tm**  **(metallated)** | **Apparent Tm**  **(demetallated with EDTA)** |
| --- | --- | --- |
| **33μm** | | |
| wtSOD1 | 75.5 ± 0.1 | 56.0 ± 0.0 |
| G93A | 68.7 ± 0.1 | 54.1 ± 0.0 |
| G37R | 69.6 ± 0.1 | 55.5 ± 0.0 |
| A4V | 70.8 ± 0.1 | 50.9 ± 0.1 |
| **11μm** | | |
| wtSOD1 | 73.8 ± 0.0 | 54.2 ± 0.1 |
| G93A | 67.8 ± 0.1 | 52.7 ±0.0 |
| G37R | 68.7 ± 0.0 | 53.7 ± 0.0 |
| A4V | 69.7 ± 0.0 | 51.4 ± 0.2 |
